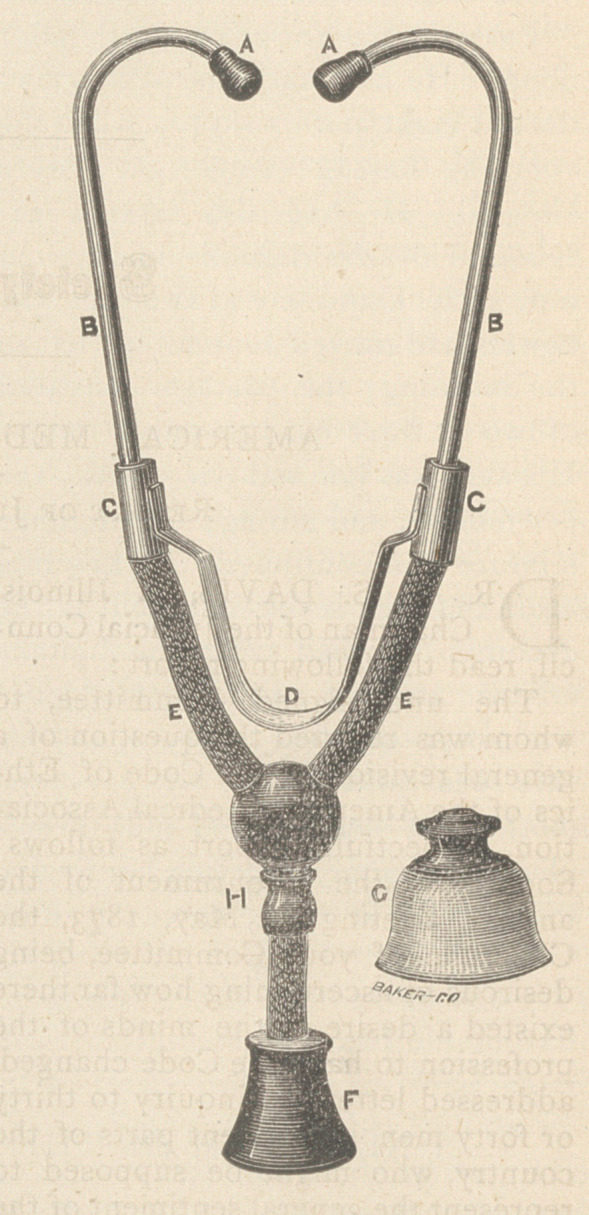# A New Form of the Binaural Stethoscope

**Published:** 1874-07-01

**Authors:** F. H. Davis

**Affiliations:** Chicago


### A NEW FORM OF THE BINAURAL STETHOSCOPE.

F. H. Davis, M.D.

THE binaural form of stethoscope, devised by Dr. Camman, from whom it takes its name, is undoubtedly a very great improvement upon the original single-barrelled wood or ivory instrument. As ordinarily manufactured, however, the Camman stethoscope possesses some objectionable features, which, we think, have detracted much from its popularity and general usefulness. In the first place, it is most inconveniently cumbersome and heavy, and not at all adapted for a pocket instrument. Now, in order to make his stethoscope at all useful as an aid to diagnosis, the physician must have it constantly by him. The particular cases and emergencies where its use is called for, are liable to occur at any and all times.

A pulmonary sound or a cardiac murmur, which is manifest but imperfectly or obscurely to the unaided ear, calls for a closer study with the stethoscope; or it may be that a case of small pox, or measles, or scarlatina is met with where pulmonary or bronchial complications are suspected, calling for a physical examination of the chest. In these latter cases we might hesitate to place our head in direct contact with the patient, and must have our stethoscope by us or omit the examination, and thus perhaps endanger the welfare of our patient. Of more frequent occurrence still, in the practice of most of us, are the cases where the filthy, uncleanly condition of the patient and his clothing renders a close contact with his person highly undesirable.

In the ordinary Camman stethoscope the fixed uniform curve of the ear tubes also prevents their proper and perfect adjustment in many cases, the same curve not suiting equally well all persons' ears. This has been a fruitful source of dissatisfaction, and has caused many physicians to condemn entirely the binaural form of stethoscope, and give preference to the old-fashioned, straight wooden tube.

Studying to overcome these imperfections, I have had manufactured for me a modified form of binaural stethoscope, a representation of which is herein appended:

Its chief peculiarity consists in the substitution of the spring D for the hinge-joint and rubber-strap which connects the tubes in the ordinary form. This gains for us several inches of room, and furnishes, besides, an easy, perfect, and much more durable adjustment to the ears. The tubes marked B disconnect from the sockets C, in packing, and can also be turned or adjusted in the same socket to the angle that best suits the ears of the one using them. The lower end and chest-piece, F, also separates at H, and the four parts of the instrument thus disconnected will pack into a case or box four and a half inches long, by three and a quarter wide, and one inch thick. This slips into the side coat pocket as easily as a memorandum book or a small pocket case.

The extra chest-piece, shown at G, has a soft rubber end, adapted to fit into the irregularities of the chest between the ribs, in emaciated subjects, where it is difficult to apply the hard end. This improvement was suggested, we believe, by Dr. Austin Flint, several years ago. Some who have used it seem to like it, others prefer the smaller hard end, which answers the same purpose.

Messrs. Shepard & Dudley, of New York, have manufactured some stethoscopes of this pattern, of very superior workmanship and finish, which are retailed at six dollars. Their agent in this city, E. H. Sargent, 785 Wabash ave., also has them on sale.

Chicago, June 20, 1874.

## Figures and Tables

**Figure f1:**